# Prenatal diagnosis and post-mortem examination in a fetus with thrombocytopenia-absent radius (TAR) syndrome due to compound heterozygosity for a 1q21.1 microdeletion and a *RBM8A* hypomorphic allele: a case report

**DOI:** 10.1186/1756-0500-6-376

**Published:** 2013-09-22

**Authors:** Irene Bottillo, Marco Castori, Carmelilia De Bernardo, Romano Fabbri, Barbara Grammatico, Nicoletta Preziosi, Giovanna Sforzolini Scassellati, Evelina Silvestri, Antonella Spagnuolo, Luigi Laino, Paola Grammatico

**Affiliations:** 1 Department of Molecular Medicine, Medical Genetics, San Camillo-Forlanini Hospital, Sapienza University, Rome, Italy; 2 Division of Radiology, San Camillo Forlanini Hospital, Rome, Italy; 3 Day Hospital – Day Surgery “Legge 194/78”, Division of Obstetrics and Gynecology, San Camillo Forlanini Hospital, Rome, Italy; 4 Service of Fetal-Neonatal Pathology, Division of Pathology, San Camillo-Forlanini Hospital, Rome, Italy; 5 Division of Obstetrics and Gynecology, ASL RMA, Rome, Italy

**Keywords:** Thrombocytopenia, Radius aplasia, 1q21.1 deletion, aCGH, RBM8A, rs139428292, TAR syndrome, Thrombocytopenia–absent radius syndrome

## Abstract

**Background:**

Thrombocytopenia–absent radius syndrome is a rare autosomal recessive disorder characterized by megakaryocytic thrombocytopenia and longitudinal limb deficiencies mostly affecting the radial ray. Most patients are compound heterozygotes for a 200 kb interstitial microdeletion in 1q21.1 and a hypomorphic allele in *RBM8A*, mapping in the deleted segment. At the moment, the complete molecular characterization of thrombocytopenia–absent radius syndrome is limited to a handful of patients mostly ascertained in the pediatric age

**Case presentation:**

We report on a fetus with bilateral upper limb deficiency found at standard prenatal ultrasound examination. The fetus had bilateral radial agenesis and humeral hypo/aplasia with intact thumbs, micrognathia and urinary anomalies, indicating thrombocytopenia–absent radius syndrome. Molecular studies demonstrated compound heterozygosity for the 1q21.1 microdeletion and the *RBM8A* rs139428292 variant at the hemizygous state, inherited from the mother and father, respectively

**Conclusion:**

The molecular information allowed prenatal diagnosis in the following pregnancy resulting in the birth of a healthy carrier female. A review was carried out with the attempt to the trace the fetal ultrasound presentation of thrombocytopenia–absent radius syndrome and discussing opportunities for second-tier molecular studies within a multidisciplinary setting.

## Background

Thrombocytopenia–absent radius (TAR) syndrome (MIM #274000) is a rare disorder occurring in ~0.42/100,000 individuals [1]. It is characterized by a reduced platelets’ number due to megakaryocytic thrombocytopenia and longitudinal upper limb defects affecting the radial ray and sparing the thumbs [2,3]. Severity of skeletal abnormalities varies from absence of radii with sparing of the thumb to virtual absence of upper limbs with or without lower-limb defects [4]. Additional manifestations include cow’s milk allergy, transient leukemoid reactions, and heart and urogenital defects [5]. The diagnosis of TAR syndrome is mostly clinical, based on the unique combination of hematological and radial defects in newborn infants and toddlers. Although TAR syndrome is not uniformly fatal, its severe morbidity and mortality could make its early prenatal diagnosis desirable. Some authors have pointed out the possibility of a prenatal diagnosis of TAR syndrome by evocative ultrasound findings [6].

In the past, different modes of inheritance have been evoked to explain recurrence of TAR syndrome, with the autosomal recessive pattern being considered most likely [3,7]. In 2007, Klopocki *et al.*[4] described a heterozygous 200 kb microdeletion at HSA 1q21.1, spanning 11 loci, in most patients. The deletion occurred *de novo* in 25% of cases and was inherited in the remaining (~2/3 from the unaffected mother, 1/3 from the unaffected father). Inheritance was clarified by the identification of low-frequency regulatory variants of the gene *RBM8A*, mapping in the deleted segment, in trans with the deletion in the affected individuals [8]. Two single nucleotide polymorphisms (SNPs) including rs139428292, located in the 5’ untranslated region (5’UTR) of *RBM8A*, and rs201779890, located in intron 1, account for the hypomorphic variants leading to reduced RBM8A transcription and protein expression [8]. These findings together with what is already known of the antenatal presentation of TAR syndrome may offer a rapid and accurate approach for diagnosing this condition in a prenatal setting.

We describe the ultrasound, pathologic and radiographic characterization of a 21-week-old fetus with a molecularly confirmed diagnosis of TAR syndrome due to co-segregation of 1q21.1deletion and rs139428292.

## Case presentation

**Figure 1:**
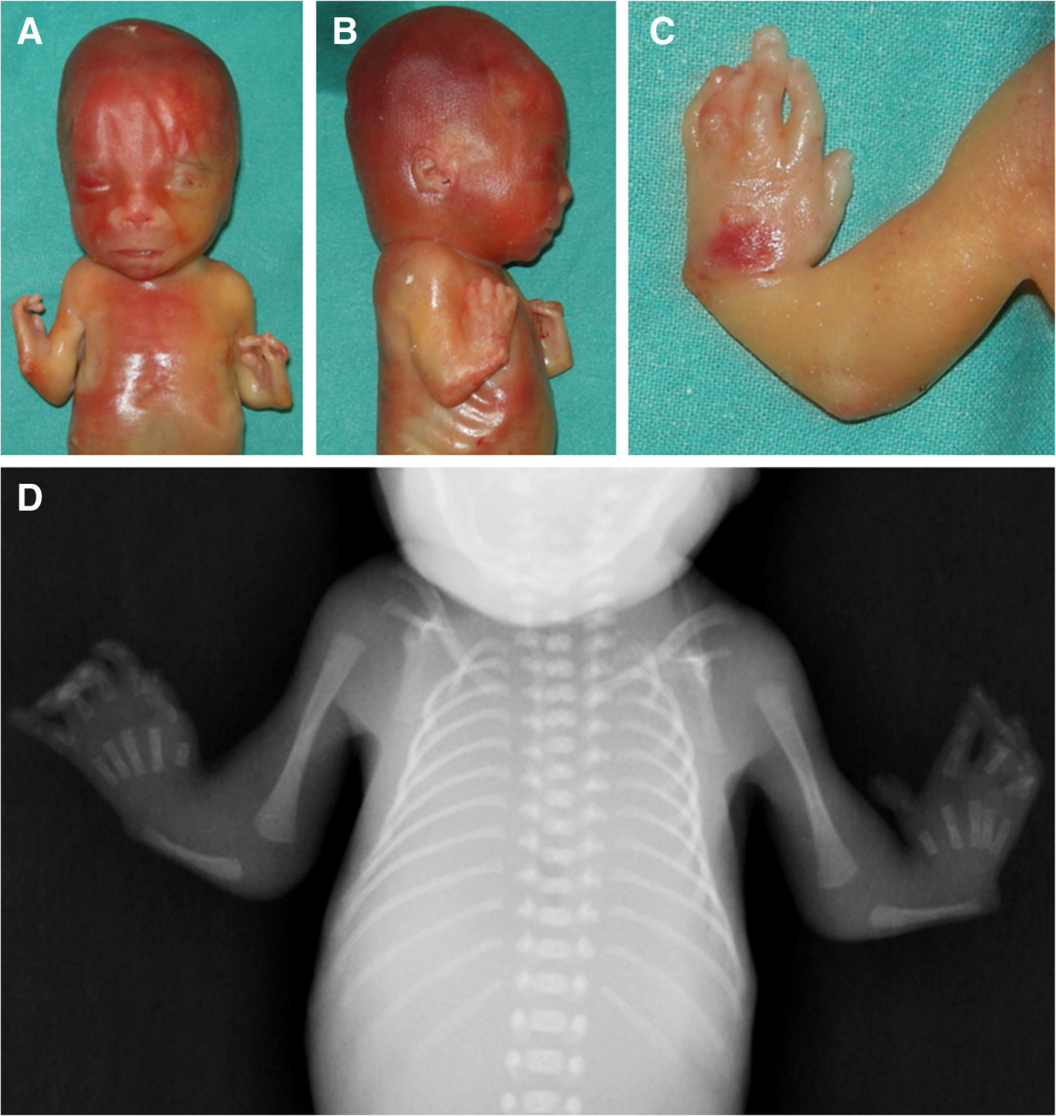
**Clinical and radiologic findings.** Frontal **(A)** and right lateral **(B)** views of the fetus showing flat nasal bridge with midly anteverted nares and receding chin. Severely shortened forearms, radial club hands and presence of thumbs. Magnification of the left hand **(C)** shows camptodactyly with bulbous ends, pterygium between wrist and the rhizomelic segment, and normally placed thumb. Babygram confirms the absence of both radii with hypoplastic and straight ulnae **(D)**. Note normal ossification of the acral segment.

The patient was the first conceptus of a 32-year-old Caucasian woman and her healthy and non-consanguineous 36-year-old husband. Early pregnancy and family history were unremarkable. Fetal standard ultrasound scan at the beginning of the 21^st^ gestation week documented micrognathia, generalized shortness of the upper limbs (<5^th^ centile) with severe hypoplasia of forearms containing a single bone and measuring 16 mm on the right and 12 mm on the left, bilateral club hands, left club foot, apparent hypospadias, borderline dilatation of the left lateral ventricle (11 mm) and bilateral pyelectasis (Table 1, Figure 1). Amniotic fluid volume was normal. Amniocentesis was not performed.

**Table 1:** Previously reported and present patients with thrombocytopenia–absent radius syndrome ascertained by prenatal diagnosis

**Characteristic**	**Patients**																			**Total**
**Reference**	[9]	[10]	[11]	[12]		[13]	[14]	[15]		[16]	[17]	[18]		[19]	[20]	[21]	[22]		Present	
Patient identification by ref.				A	B			1	2			2	3				4	12		
Previously affected child	+	+	+	+	+							+	+							6/11
Maternal age (years)	28	29	25	35	35	18		30	30	33	22			21	33	36			31	
Gestational age (weeks)	20	19	17	19	19	32	23	25	32	18	38	18	13	MP	16	13	13	13	21	
Sex			F	M	F	M	M	F	F	M	F	F	F	M			F	F	M	9F, 5M
IUGR	+					+			+											3/12
Thrombocytopenia						+	-	+^1^	+	+^2^		+		-	+					6/8
Upper limbs anomalies	+	+	+	+	+	+	+	+	+	+	+	+	+	+	+	+	+	+	+	19/19
Humeral hypo/aplasia						+	+												+	3/3
Short forearms			+					+^1^											+	3/3
Radial hypo/aplasia	+	+			+	+	+	+	+	+		+	+	+	+	+	+	+	+	16/16
Ulnar hypo/aplasia	+	+				+	+					+				+			+	7/7
Club hands		+		+	+			+		+		+	+		+	+			+	10/10
Lower limbs anomalies						+	+			-								+		4/5
Other features			CH	OH					AH, R, VM						PH		CH		Py, VM	

^1^: observed at 32 weeks. ^2^: obsterved at 37 weeks.

*IUGR* intrauterine growth restriction, *bil* bilateral, *A* aplasia, *Ab* spontaneous abortion, *AH* aortic hypoplasia, *CH* cystic hygroma, *H* hypoplasia, *OH* oligohydramnios, *MP* mid-pregnancy, *PH* polyhydramnios, *Py* pyelectasis, *RD* renal dysplasia, *ToP* termination of pregnancy, *VM* ventriculomegaly.

External examination of this male fetus at 21^st^ gestation week showed severe shortness of the forearms with radial club hands, moderate shortness of the rhizomelic segment of the upper limbs, flat face with anteverted nares and a receding chin. Flexion contractures and bulbous ends of the fingers were observed at both hands which showed well-formed thumbs. Lower limbs and trunk were unremarkable. The fetus weighted 265.7 g, was long (crown-heel length) 25.5 cm and had a head circumference of 19 cm. Internal examination demonstrated multiple, small bilateral kidney cysts with mild dilatation of the pelvis and megacystis due to stenosis of the lower urinary tract. Hypospadias was not confirmed. At total-body X-ray study noted bilateral absence of the radii and hypoplasia of ulnae and homeri (Figure 1). The clinical diagnosis of TAR syndrome was established and molecular testing was performed.

**Figure 2:**
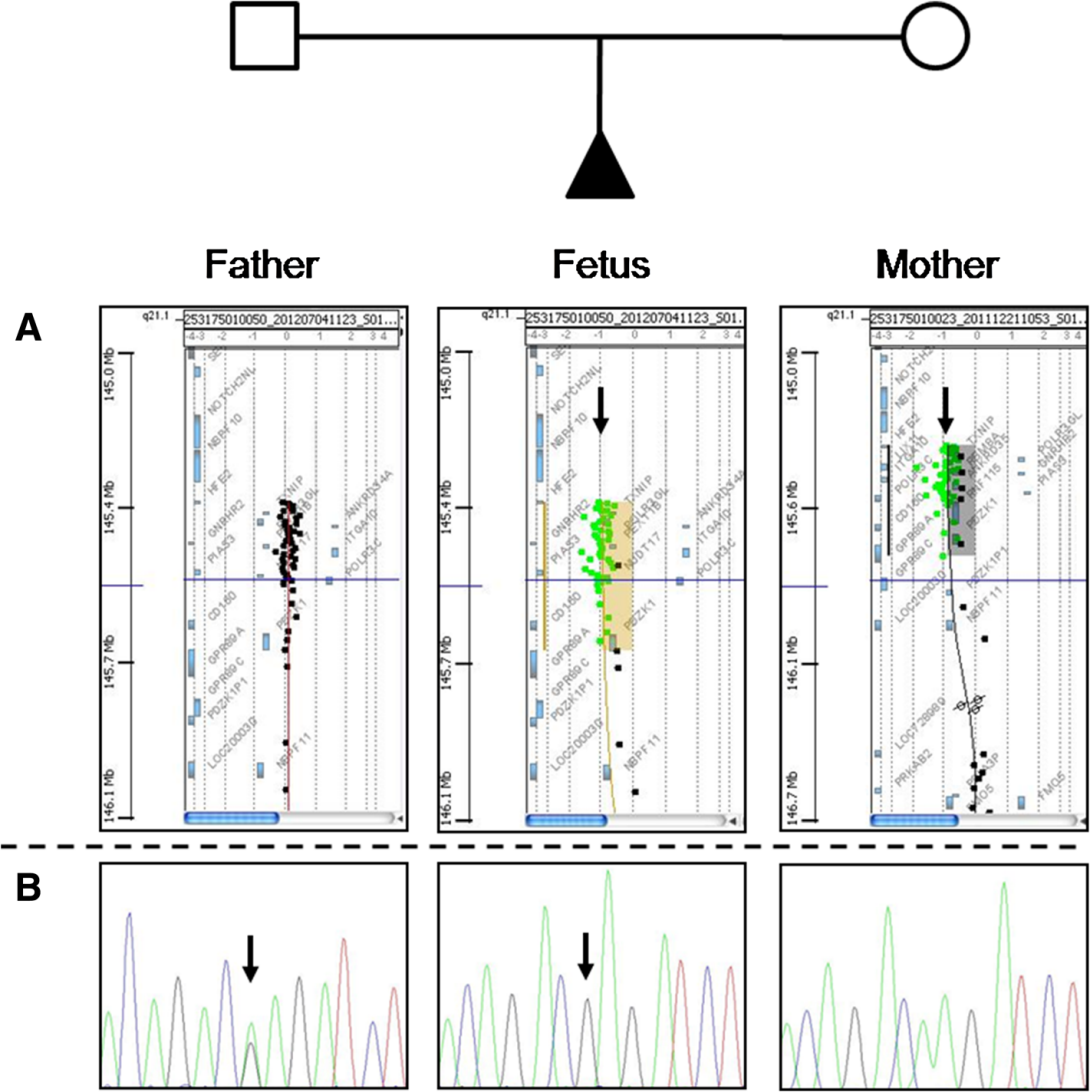
**Molecular characterization of the pedigree. Panel A**: aCGH profile of the nuclear family indicating normal genomic content in the mother at 1q21.1 region, while in the fetus and father the typical deletion is present (black arrows). **Panel B**: sequencing of *RBM8A* at rs139428292 showing the minor A allele at the heterozygous state in the mother and at the homo(hemi)zygous state in the fetus (black arrows), while the father is homozygous for the major G allele.

The couple gave informed consent for DNA (deoxyribonucleic acid) analysis, which was approved by local ethic committees in accordance with the guidelines of SIGU (Italian Society of Human Genetics) and recent law concerning genetic data published in “G.U. n.159 11-07- 2011”. Genomic DNA was extracted from the fetus and parental peripheral blood by standard methods. The DNA of the patient was first analyzed by using the commercially available Human Genome CGH (Comparative Genomic Hybridization) Microarray Kit 2x105K (Agilent Technologies, Santa Clara, USA). Labeling and hybridization of patient’s DNA, and pooled male and female control DNAs were performed according to the manufacturer’s protocol. Further analyses and visualization were performed with “Agilent Genomic Workbench version 6.5”. The fetus was found to carry a ~390Kb heterozygous deletion in 1q21.1 which spanned from 145415190bp to 145804738bp (UCSC version GRCh37/hg19, released in February 2009). Genomic DNA of both parents was analyzed in order to determine the parental origin of the deletion, which was subsequently identified in the healthy mother. Genotyping of rs139428292 (G>A) and rs201779890 (G>C) SNPs was carried out by direct sequencing. The following primers were used for polymerase chain reaction (PCR) amplification: Fw-5’- GCCTTTGATTGGTCAGCTTG-3’ (located in the *RBM8A* 5’UTR) and Rv-5’- AAGGGGGCGGAATCTCTAAT-3’ (located in *RBM8A* intron 1). Forward and reverse sequences were analyzed and compared with the messenger ribonucleic acid (mRNA) reference sequence (NM_005105). The analysis of rs139428292 showed that the fetus harbored the minor A allele, which was inherited from the healthy father (Figure 2). All of them carried the major G allele of rs201779890 in a homozygous state. This information allowed prenatal diagnosis in the following pregnancy of the couple with the identification of a healthy fetus heterozygous carrier of the paternal allele.

## Discussion

Table 1 summarizes the present patient and 18 previously reported cases of TAR syndrome ascertained prenatally. All fetuses were investigated antenatally by ultrasound scan. Three cases [9,10,12] also underwent fetal radiography, followed by fetoscopy in one [12]. Cordocentesis was performed in 7 patients [13,15,16,18-20] and led to results compatible with thrombocytopenia in 6 of them [13,15,16,18,20]. Genomic copy number imbalances were investigated in 5 cases by array-CGH (aCGH) technique [18,22]. Case 4 and case 12 by Houeijeh and co-workers were found to carry respectively a 333–774 kb and a 671–938 kb *de novo* heterozygous deletion, both mapping to HSA 1q21.1 [22]. In case 12 of the same series, inheritance of the rearrangement was not investigated [22]. The mother of fetuses 2 and 3 reported by Boute *et al.*[18] was found to carry a genomic heterozygous deletion in 1q21.1 [22]. No patient, except the present one, was further investigated for *RBM8A* nucleotide variations. All upper limb malformations reported in Table 1 were bilateral.

The present family increased to 56 the number of cases with TAR syndrome fully characterized at the molecular level (i.e. aCGH *plus RBM8A* molecular screening) and, among them, our case was the first ascertained prenatally. Recognition of this fetus occurred by ultrasound detection of radial clubhands and longitudinal defects of both forearms which were constituted of a single bone, an association suggestive of bilateral radius agenesis. Prognosis is difficult in case of prenatal occurrence of radial defects. A literature review showed that the typical prenatal presentation of TAR syndrome consists of bilateral radial hypoplasia/agenesis with or without humeral shortness, and p resence of thumbs on both hands. In this setting it is hence suggested to proceed to invasive diagnosis with the primary aim of excluding a 1q21.1 microdeletion followed by *RBM8A* mutational screening. A 1q21.1 microdeletion may go unnoticed at the standard resolution of aCGH (usually, 200Kb – 1Mb). Once the rearrangement is identified, extension to *RBM8A* testing is crucial not only to confirm the diagnosis, but also for accurate recurrence risk calculation and monitoring of future pregnancies, as occurred in the present family.

## Conclusions

Our findings confirm the genetic homogeneity of TAR syndrome and the existence of recurrent mutations for this condition. The minor allele frequency of the 5’UTR and intronic *RBM8A* SNPs is 3.05% and 0.42%, respectively in 7,504 healthy individuals of the Cambridge BioResource [8]. That rate of occurrence may explain the unusual inheritance pattern in non-consanguineous families: vertical parent-to-child transmission [23], as well as the case of a male patient and maternal uncle [24]. The frequency of the TAR deletion (1/8329, [25]) and the frequency of two noncoding SNPs in European population are roughly consistent with the incidence of 1:240.000 [1] and with the ratio of affected to unaffected deletion carriers [4]. Both breakpoints of TAR 1q21.1 deletion were found to be enriched for low-copy repeats [4] that could drive the occurrence of the chromosomal alteration. Resolution of the TAR syndrome genetics will allow couples with previously affected fetus(es)/child(en) to monitor future pregnancies and to extend awareness to other relatives and their unrelated partners at risk of being carriers of *RBM8A* predisposing polymorphisms.

## Consent

Written informed consent was obtained from the parents of the fetus for publication of this Case Report and any accompanying images. A copy of the written consent is available for review by the Editor-in-Chief of this journal.

## Abbreviations

TAR: Thrombocytopenia–absent radius; SNP: Single nucleotide polymorphisms; DNA: Deoxyribo nucleic acid; CGH: Comparative genomic hybridization; 5’UTR: 5’UnTranslated region; aCGH: array-CGH; IUGR: Intrauterine growth restriction; Bil: Bilateral; A: Aplasia; Ab: Spontaneous abortion; AH: Aortic hypoplasia; CH: Cystic hygroma; H: Hypoplasia; OH: Oligohydramnios; MP: Mid-pregnancy; PH: Polyhydramnios; Py: Pyelectasis; RD: Renal dysplasia; ToP: Termination of pregnancy; VM: Ventriculomegaly.

## Competing interests

The authors declare that they have no competing interests.

## Authors’ contributions

BI participated in the design and coordination of the study, carried out the molecular genetic studies and drafted the manuscript; CM provided the genetic counseling to the patients, established the clinical diagnosis, participated in the study design and helped to draft the manuscript; DBC participated in the design and helped to draft the manuscript; FR performed the radiographic characterization of the fetus and gave the final approval of the version to be published; GB participated in the design and helped to draft the manuscript; PN participated in the design of the study and carried out the molecular genetic studies; SSG conceived of the study and gave the final approval of the version to be published; SE performed the pathologic examination of the fetus and gave the final approval of the version to be published; SA performed the ultrasound scan and gave the final approval of the version to be published; LL participated in the design of the study, carried out the molecular cytogenetic studies and gave the approval of the version to be published; GP conceived of the study and gave the final approval of the version to be published. All authors read and approved the final manuscript.
